# Jumping Genes Reveal Kangaroos' Origins

**DOI:** 10.1371/journal.pbio.1000437

**Published:** 2010-07-27

**Authors:** Mason Inman

**Affiliations:** Freelance Science Writer, Karachi, Pakistan

**Figure pbio-1000437-g001:**
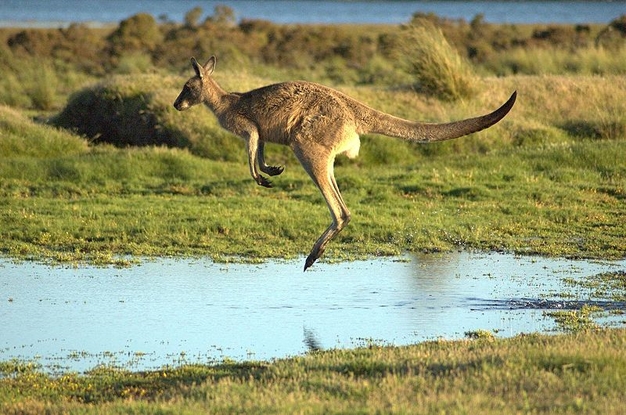
Comparative genomic analysis of retrotransposons, also known as jumping genes, helps refine the long-debated marsupial family tree, placing the ancient roots of Australia's trademark kangaroo and its evolutionary relatives in South America. (Image: PanBK/Wikimedia)

[Fig pbio-1000437-g001]Nothing is more Australian than kangaroos. But these marsupials—along with a variety of others including the Tasmanian devil—have ancient roots in South America, a new study suggests.

Exactly how these various marsupials, both living and extinct, are related has been murky. There are marsupials found today in both Australia and the Americas, with the opossum the most familiar to Americans.

Some older studies suggested that marsupials first arose in Australia and that some marsupial lineages might have been split in two when these landmasses separated 80 million years ago. But there are few fossils from either South America or Australia of long-extinct marsupials, so debates have raged for decades about how to arrange the branches of the marsupial family tree. Genetic studies—looking both at genes in cells' nuclei and in mitochondria, the cells' powerhouses that carry their own DNA—have come up with contradictory results about which lineages are most closely related and which split off first.

In this issue of *PLoS Biology*, Maria Nilsson, Jürgen Schmitz, and colleagues present the first study to use the sequences of retroposed elements—a kind of “jumping gene”—to reconstruct marsupials' family tree. The new results may finally give a clear answer as to where marsupials arose and how they branched off from each other; but their findings may surprise some researchers, as they're at odds with some earlier work.

Retroposed elements make up a bigger portion of kangaroos' and other marsupials' genomes than any other mammal that's had its genome sequenced. The sequences appear to serve little or no purpose to these animals, but that's exactly what gives the new technique its strength.

Retroposons use their own enzymatic machinery, or that of other retroposons, to copy their own RNA and create DNA copies of themselves. Instead of making copies to spread from cell to cell and organism to organism, as, for example, viruses do, retroposed elements are deposited in other parts of the same genome in the same cell—including in the germ line cells—cutting a gap in a DNA strand and inserting themselves there. These copies remain in their new locus. It is extremely rare that a retroposed element is cleanly excised sometime after insertion. Afer millions of years, hundreds of thousands of them are now littered throughout marsupials' genomes.

Also, the way they spread through the genome means they can occur in idiosyncratic patterns. Jumping genes are so widespread in marsupial genomes that when a copy jumps, it often lands in the middle of an older jumping gene, creating one retroposon nested within another one. Retroposons, and especially nested ones, are unlikely to arise independently in another species in exactly the same part of the genome by chance. So if different species share a few of the same nested retroposed elements, chances are overwhelming that they all got them from a long-lost ancestor.

In two marsupial genomes that were recently sequenced, Nilsson, Schmitz, and colleagues identified thousands of these nested retroposed elements—more than 8,000 in the genome of the South American opossum (*Monodelphis domestica*), and nearly 4,500 in the genome of a kangaroo, the Australian Tammar Wallaby kangaroo (*Macropus eugenii*).

Nilsson, Schmitz, and colleagues sorted all these nested retroposed elements into three categories: those unique to the kangaroo, those unique to the opossum, or those shared between the two. They pared down the thousands of jumping elements to 53 that would serve as markers of how various marsupials branched off from each other. They found that all living marsupials must have come from one branch of mammals, since they all share jumping elements in 10 particular spots in their genomes, which are not found in any other mammals.

They then searched through the DNA of 20 marsupial species—including the wallaroo, the common wombat, and the marsupial mole—to see which of these markers they carry. It hadn't been clear which lineage of marsupials split off first, but the new study found this first branch gave rise to the Didelphimorphia lineage, which includes several species of opossums of South America. Further branches gave rise to other South American marsupials—the Paucituberculata, or shrew opossums, and Microbiotheria, which includes only one remaining species, the Monito del Monte, or “little mountain monkey” (*Dromiciops gliroides*).

All of the today's Australian marsupials appear to have branched off later, all arising from a single lineage that branched from a South American microbiotherian-like ancestor to form varied forms—kangaroos, the rodent-like bandicoots, and the Tasmanian devil.

It's still a bit of a mystery, Nilsson, Schmitz, and colleagues say, why the marsupial family tree sorted out so cleanly. They found two distinct branches—one for South America and one for Australia—despite these landmasses having formed parts of the larger landmass of Gondwana for tens of millions of years around the time that marsupials arose. Regardless, the new study argues, this new family tree should help researchers make sense of other genetic markers and features of skeletons that had led to contradictory results before. Jumping genetic elements are themselves shifty, but they could help settle the science.

**Nilsson MA, Churakov G, Sommer M, Tran NV, Zemann A, et al. (2010) Tracking Marsupial Evolution Using Archaic Genomic Retroposon Insertions. doi:10.1371/journal.pbio.1000436**

